# Exploring the Dynamics of Caring for a Child With a Terminal Illness of Duchenne Muscular Dystrophy (DMD) and Its Copious Components on the Caregivers

**DOI:** 10.7759/cureus.39597

**Published:** 2023-05-28

**Authors:** Adelina Balidemaj, Parmis Parsamanesh, Mykhailo Vysochyn

**Affiliations:** 1 Clinical Sciences, Saint James School of Medicine, Chicago, USA; 2 Cardiology, Saint James School of Medicine, The Valley, AIA

**Keywords:** family burden/care burden, parents caregivers, muscular dystrophy, psychological well-being, mental health, dmd, duchenne muscular dystrophy

## Abstract

Duchenne muscular dystrophy (DMD) is an inherited disorder that results in increasing muscle degeneration and muscle weakness because of a mutation in the dystrophin protein. Despite there being no cure for this condition, early diagnosis can slow down the progression of muscle weakness. Studies have shown that families and caregivers of patients with DMD have limited access to support systems, further intensifying their responsibilities. Since the mental well-being of families and caregivers of patients with DMD is crucial for healthy and progressive family dynamics, ascertaining the psychological and social impacts it has on the caregivers will help improve the quality of life of patients with this terminal illness.

This study aims to recognize the direct and indirect effects on the caregiver caring for the individual diagnosed with DMD, with a primary focus on the health-related quality of life (HRQoL), psychological well-being, and financial burden.

Using the PubMed database and a particular arrangement of Medical Subject Headings (MeSH) terms, 93 articles were retrieved and evaluated, of which eight only met the inclusion criteria. The eight chosen articles were organized into a table and dissected further for their importance and relevance to this review article. This literature review highlights the most significant information from each article and is individually analyzed to deduce the main burdens on caregivers of patients with the terminal illness of DMD.

Conclusively, this review highlights that caregivers of individuals with DMD bear a substantial burden that negatively affects their HRQoL, contributes to a decline in psychological well-being, and places an increased financial burden on the family.

## Introduction and background

Duchenne muscular dystrophy is a genetic disorder resulting from a mutation of the DMD gene located on the short arm of the X chromosome (Xp21.2) [1-6]. It is a prevalent form of muscular dystrophy leading to increasing muscle weakness. Currently, there is no cure for DMD [1]. This mutation on the DMD gene affects the production of dystrophin, leading to an absence or insufficient amount of protein necessary for maintaining the structure and function of muscles. Dystrophin plays a role in keeping muscle cells intact [1].

This is particularly prevalent when assessing motor or movement developmental milestones. Symptoms of DMD often begin when a child is between the ages of two to five years old [5]. Affected boys with DMD start walking later than their peers, and often it is thought that they are clumsy and lack coordination. Depending on the severity of DMD, other symptoms can include learning difficulties (IQ below 75), fatigue, and muscle weakness beginning in the legs and pelvis before advancing to the arms, neck, and other areas of the body. There is often difficulty with motor skills and fine dexterous movements, frequent falls, trouble getting up or climbing stairs, progressive weakness, and complete loss of ability to walk by age 12 (this can vary). As the patient becomes more sedentary, breathing difficulties are often faced, and the muscles of the chest, upper extremities, and diaphragm become weaker. Cardiomyopathy usually presents within the patient's second decade of life. Depending on the degree and nature of the DMD, these age-related milestones can be fluid, and some patients can go without experiencing all these symptoms. In contrast, others, unfortunately, are afflicted more severely [2]. 

The physical, mental, and social well-being of patients with DMD is well-recognized and commonly known. However, many of these impacts on the caregivers, particularly the parents of DMD patients, often go unnoticed and overlooked. It has been shown that providing informal care can severely affect the caregiver's health, including anxiety, depression, social isolation, and financial deprivation [4,7,8]. Resources and documents outlining the prevalent effects and impacts DMD can have on the family need to be identified and investigated. DMD is a complex and unique disease that carries challenges and adaptations prior to the diagnosis of the disorder all through to the termination of life due to complications arising directly and indirectly from DMD. These challenges are not insurmountable and negatively affect both the patient and their family and their relationships with others. Difficulties can arise in the accessibility of appropriate healthcare services and family resources [9]. Quality of life is measured by the overall satisfaction of one's life, personal growth and development, physical well-being, etc. [3]. There have been research studies that have focused on the effects terminally ill patients have on their caregivers and some that have focused on parents of children with DMD. The review aims to comprehend the direct and indirect impacts of the caregivers of individuals diagnosed with Duchenne muscular dystrophy with an emphasis on their health-related quality of life, psychological well-being, and financial burden. 

This article was previously presented as a meeting abstract at the 2022 Remote Health Monitoring Meeting on July 29, 2022.

Materials and methods

Search Protocols

The articles were searched using the PubMed (Medline) database, and the Medical Subject Headings (MeSH) combination of terms used were Duchenne muscular dystrophy [ti] AND caregivers [tw] AND impact [tw]. Solely English-written articles were retrieved.

Inclusion and Exclusion Criteria

For this literature review, 93 articles related to DMD and its impact on caregivers were retrieved. As these articles were being reviewed, the following strategies of inclusion and exclusion criteria were implemented: a title and abstract exclusion was conducted to rule out articles that did not have any association with the quality of life and impact on caregivers, articles with no study data provided, and articles that focused on treatment/management of DMD. In doing this technique, 80 articles were eliminated, which narrowed the remaining results to 13 articles. The rest of the articles were analyzed to follow the inclusion criteria. The inclusion criteria consisted of articles published between the years 2012- 2022, interventional measures of the quality of life and the impact of caregivers on people with DMD, articles written as case-control studies/ literature reviews, and articles that provided data. This method properly narrowed the articles to be evaluated for this literature review. 

Synthesis of Information and Management of Search Results

Each author reviewed the articles independently to assess if all inclusion criteria were met. Those that met the inclusion criteria were compiled into the tables for the observation of data. The impacts on the caregivers include health-related quality of life, specifically their psychological well-being, financial burden, and mental health status. Because of the nature of this review, no experimental equipment or materials were necessary.

## Review

Results

**Figure 1:**
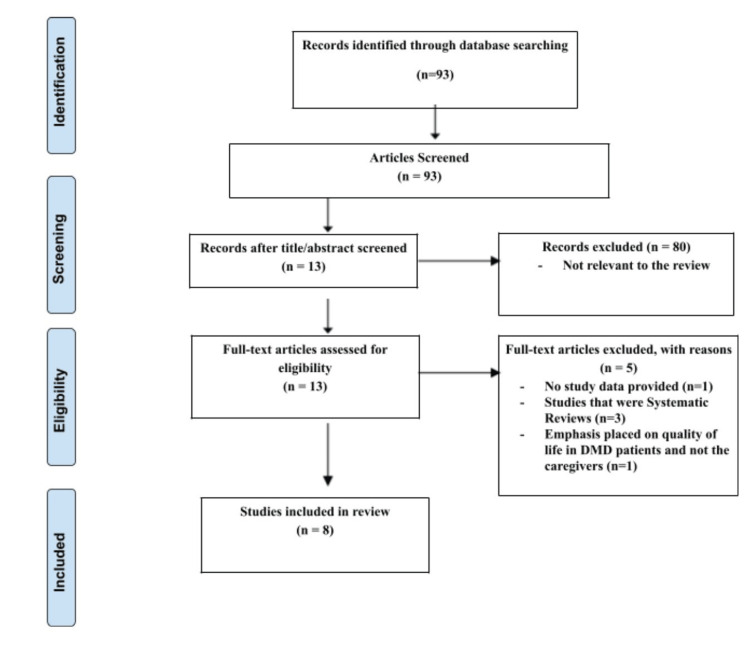
Preferred Reporting Items for Systematic Reviews and Meta-Analysis

The search strategy yielded 93 articles. Eighty articles were excluded based on the title and abstract of each article that needed to have relevance to this literature review. The remaining 13 articles were analyzed for eligibility based on the inclusion criteria. As the remaining articles were being reviewed, five articles were excluded because one article did not provide any data associated with the study, three were systematic reviews, and one focused on the quality of life in DMD patients and not the caregivers. Therefore, based on the inclusion criteria, this review included eight articles dealing with the interventional measure of the quality of life, financial burden, and psychological well-being of caregivers to people with DMD, published from 2012-2022. The process of obtaining these articles is shown in Figure 1.

During the review of the eight articles, six included data relevant to health-related quality of life and psychological well-being. The findings of these articles agreed that DMD caregivers experienced a great negative impact on daily activities, physical health impairment, exhibited feelings of anxiousness and depression, diminished social life, and impairment in relationships. Detailed observations are shown in the tables provided within this paper, which specify the participants, methods for data collection, main aspects investigated, instruments used, and main findings.

Five articles included data relevant to the financial burden of DMD-related expenses in a family household caring for a DMD child. In those articles, there was an agreement that the higher-impact families experienced a great deal of financial burden. Out-of-pocket expenses consisted of home renovations required to be accessible to non-ambulatory affected DMD children, which created difficulty in paying bills and a financial burden. Detailed observations are shown in the tables provided within this paper, which specify the participants, methods for data collection, main aspects investigated, instruments used, and main findings.

Discussion

Health-Related Quality of Life

There is compelling evidence that demonstrates the health-related quality of life of the caregivers of individuals with DMD significantly decreased over time. In particular, as the illness progresses and patients become ambulatory or require ventilation support, the caregiver's responsibilities increase, and they begin allocating more time to the patient. The patient's muscles slowly deteriorate, becoming limited to basic daily tasks such as using the bathroom, lying down, eating, etc. Not only are the DMD patients trying harder than the average individual to get simple tasks done, but the caregivers of such patients endure a lot of mental, emotional, and physical stress to assist at all times. Therefore, parents or family members who take the role of a caregiver must learn to handle a child's DMD diagnosis, how symptoms arise and advance rapidly, and that this illness is terminal, resulting in the eventual loss of their loved ones. Thus, over time, the physical, emotional, mental, and social functioning of the caregivers deteriorates [4, 5, 6]. The articles pertaining to health-related quality of life are organized in Table 1.

**Table 1:** Health-related quality of life and psychological well-being DMD - Duchenne muscular dystrophy, COIDUCH - cost of illness in patients with Duchenne muscular dystrophy, COI - cost of illness, HRQoL - health-related quality of life, WPAI: GH - work productivity and activity impairment: general health, USNIH - United States National Institutes of Health, PAG and MAXQDA - program analysis group and max qualitative data analysis

Study	Setting; sample	Methods for data collection	Main aspects investigated	Instruments	Main findings
Quality of Life and Informal Care Burden Associated With Duchenne Muscular Dystrophy in Portugal: The COIDUCH Study [1]	46 DMD caregivers (8 ambulatory, 38 non-ambulatory patients)	Caregivers to patients with DMD registered through the Portuguese Neuromuscular Association; face-to-face interviews with trained interviewers followed by caregivers answering a customized questionnaire.	COI, HRQoL, the burden on caregivers specifically related to missed work hours, reduced performance at work, overall impact at work (combined missed work hours and impairment while working), and impairment in daily activities.	EuroQol-5D (EQ-5D-3L), WPAI: GH, Wilcoxon, and Kruskal-Wallis tests.	The average daily impact on activities was 60%. An average daily activity impairment of 68% was showcased in caregivers taking care of non-ambulatory individuals. Caregivers of non-ambulatory individuals who require either full-time or part-time ventilation support had a greater daily activity impairment of 77% compared to those individuals without ventilation support, whose impairment was 57%. Significant difficulties were noted in self-care, mobility, and activity. Additionally, work performance was reported to be impaired at 30.5%.
Quantifying the Burden of Caregivers in Duchenne Muscular Dystrophy [4]	In the multinational study, 770 caregivers	Caregivers to eligible patients were invited to complete a questionnaire online.	To explore the subjective caregiver burden corresponding with DMD, the prevalence of anxiety and depression, and caregiver health-related quality of life.	EuroQol EQ-5D-3L (EQ-5D), a visual analog scale (VAS), and the SF-12 Health Survey (SF-12) [4].	Approximately half of the caregivers reported being moderately or extremely anxious or depressed (p<0.001) when compared to the general population. A large proportion of DMD caregivers reported having pain or discomfort and problems performing usual activities. Nearly 30% of caregivers estimated the annual household cost burden at over $5000 (p<0.006).
Characterizing the Quality-of-Life Impact of Duchenne Muscular Dystrophy on Caregivers a Case-Control Investigation [7]	566 DMD caregivers, 594 non-DMD comparison caregivers	A web-based survey included DMD caregivers and a nationally representative comparison group of parents of children without DMD stratified by child age group	To examine the impact of DMD on family member caregivers in terms of QOL, life stress, and indirect costs.	PROMIS-10 General Health, NeuroQOL Positive Affect and Well-Being, The Ryff Environmental Mastery, and the Centers for Disease Control (CDC) Healthy Days Core Module.	The caregivers of patients with DMD reported poorer physical and mental health, positive affect, environmental mastery, and stressful life events and faced greater difficulties in paying bills compared to other caregivers.
Drivers of Caregiver Impact in Duchenne Muscular Dystrophy: A Cohort Study [9]	566 DMD caregivers	Eligible participants were age 18 or older, able to complete an online questionnaire, and were providing caregiving support to a family member with DMD at least two years old, usually their son.	The association within various psychosocial factors and impact profiles among selected DMD caregivers. The psychosocial factors analyzed incorporated demographic, QOL, life stress, resilience, COVID-related, reserve-building, and cognitive appraisal approach.	Person-reported outcomes (PROs), PROMIS-10 General Health, NeuroQOL Positive Affect and Well-Being, The Ryff Environmental Mastery, Urban Life Stress Inventory, Work Productivity and Activity Impairment measure, USNIH, The CDC Healthy Days Core Module, Current-Reserve-Building Measure, QOL appraisal, and ANOVA.	As the child's disability progresses, the impact experienced by DMD caregivers varies depending on their personal health, environmental mastery, and levels of anxiety. Caregivers who were more significantly affected experienced a worsened impact during the COVID pandemic. Additionally, engagement in passive-media consumption and a cognitive pattern focused on the negative were associated with a worsened impact for caregivers [9]. Stress variables from the high-impact group revealed that difficulty paying bills and hours missed from work was most prominent and worsened compared to the low-impact group.
Measuring Duchenne Muscular Dystrophy Impact: Development of a Proxy-Reported Measure Derived From PROMIS Item Banks [10]	521 DMD caregivers	A web-based study following telephone interviews as a pretest	Fatigue support, strong impact, cognitive function, upper extremity function, positive affect, negative affect, sleep-device symptoms, and mobility.	PROMIS item banks	Caregivers presented with comorbid health conditions, with the most prevalent being back pain, depression, insomnia, and arthritis.
A Qualitative Study on the Impact of Caring for an Ambulatory Individual With Nonsense Mutation Duchenne Muscular Dystrophy [11]	10 DMD caregivers	Patients with DMD from Germany, Italy, the UK, and the US were recruited through national registries. Caregivers are invited to complete an online questionnaire.	The impacts and challenges experienced by caregivers of ambulatory individuals before treatment with ataluren.	PAG and MAXQDA	There were proximal impacts reported by the caregivers such as physical, emotional, and distal influences that affected the dynamic of their social life, work, and relationships [11]. Disrupted sleep patterns were present due to care for the DMD child at night. Several caregivers experienced grief/sadness upon their child's diagnosis and watched them deteriorate. Caregivers were diagnosed with depression., experienced anxiety and a high load of stress. After Ataluren, DMD patients experienced positive changes that had direct impacts on caregivers. DMD caregivers were less anxious, their back pain subsided, increased their social life, able to go to work, and overall a positive impact of ataluren on their quality of life because they could see their son improve.

Psychological Well-Being

The psychological well-being of caregivers is an important component to analyze as their mental health has a direct effect on their day-to-day activities and responsibilities. Studies have revealed the psychological burden notably increased as the child with DMD became non-ambulatory. In a 2016 study titled "Quantifying the Burden of Caregiving in Duchenne Muscular Dystrophy", anxiety and depression were recorded in approximately 70% of caregivers [5]. Such anxiety stems from the economic burden, social limitations, and great time devoted to caring for a patient with DMD. Being a caregiver while having the mental load of this important role takes a toll on their efficiency to focus, stay motivated, and remain positive in life. More specifically, if the caregiver is self-dependent and has no external help or anyone to speak to about the challenges they encounter daily, being negatively affected mentally is inevitable. Therefore, the amount of time caregivers put on their patients over time takes away time for themselves, creating challenges when coping with their mental health. The articles pertaining to psychological well-being are organized in Table 1.

Financial Burden

There are many costs associated with the care of a patient with DMD, which can create a financial burden. A study from 2014, titled "The Burden of Duchenne Muscular Dystrophy", elaborated on the costs associated with informal care, healthcare resource use, and production loss for caregivers from different nations (Germany, Italy, the United Kingdom, United States) [6]. Studies indicate that DMD carries a large economic burden that increases with disease progression. In particular, the patients require various medical assistance throughout their lifetime alongside other personal expenses such as a new wheelchair for different stages of life (the sizing of a wheelchair depends on the age of the patient) and associated gadgets. The calculation of the household burden of DMD considered various factors, including income loss, the monetary value of lost leisure time, and reduced quality of life (intangible costs), estimated at between $58,440 and $71,900. As a result, it is vital to consider the costs of providing care for a patient with DMD and how it can ultimately lead to a substantial economic burden [7, 8, 9]. The articles pertaining to the financial burden are organized in Table 2.

**Table 2:** Financial burden DMD - Duchenne muscular dystrophy, COIDUCH - cost of illness in patients with Duchenne muscular dystrophy, COI - cost of illness, HRQoL - health-related quality of life, WPAI: GH - work productivity and activity impairment: general health, USNIH - United States National Institutes of Health, PAG and MAXQDA - program analysis group and max qualitative data analysis

Study	Setting(s); sample	Method(s) for data collection	Main aspect(s) investigated	Instrument(s)	Main finding(s)
Quantifying the Burden of Caregivers in Duchenne Muscular Dystrophy [4]	In the multinational study, 770 caregivers	Caregivers to eligible patients were invited to complete a questionnaire online	To explore the subjective caregiver burden corresponding with DMD, the prevalence of anxiety and depression, and caregiver health-related quality of life.	EuroQol EQ-5D-3L (EQ-5D), a visual analog scale (VAS), and the SF-12 Health Survey (SF-12) [4].	Approximately half of the caregivers reported being moderately or extremely anxious or depressed (p<0.001 )when compared to the general population). A large proportion of DMD caregivers reported having pain or discomfort and problems performing usual activities. Nearly 30% of caregivers estimated the annual household cost burden at over $5000 (p<0.006).
The Burden of Duchenne Muscular Dystrophy [5]	770 patient-caregiver pairs; Germany: 173, Italy: 122, United Kingdom: 191, United States: 284	Eligible patients and one of their caregivers (e.g., parent) were invited to complete a questionnaire online	To estimate the overall cost of illness and the economic burden imposed by DMD on both society and caregiver households.	Health Utilities Index and EuroQol EQ-5D	The overall societal burden was estimated to range between $80,120 and $120,910 per patient and increased as the disease progressed [5]. The average annual household burden was estimated to be between $58,440 and $71,900 [5].
Characterizing the Quality-of-Life Impact of Duchenne Muscular Dystrophy on Caregivers a Case-Control Investigation [7]	566 DMD caregivers, 594 non-DMD comparison caregivers	A web-based survey included DMD caregivers and a nationally representative comparison group of parents of children without DMD stratified by Child Age Group	To examine the impact of DMD on family member caregivers in terms of QOL, life stress, and costs that incur indirectly.	PROMIS-10 General Health, NeuroQOL Positive Affect and Well-Being, The Ryff Environmental Mastery, and the Centers of Disease Control (CDC) Healthy Days Core Module.	The caregivers of patients with DMD reported poorer physical and mental health, positive affect, environmental mastery, and stressful life events, and faced greater difficulties in paying bills compared to other caregivers.
Interplay of Disability, Caregiver Impact, and Out-of-Pocket Expenditures in Duchenne Muscular Dystrophy: A Cohort Study [8]	566 DMD caregivers	A web-based survey	The relationship between caregiver impact domains and out-of-pocket expenditures, as well as the presence of clusters in caregivers based on DMD-related disability domains in the patients they provided caregiving support for.	DMD caregiving Impact Measure, PROMIS-derived parent-proxy (PPP), Cohen's criteria, Hierarchical cluster analysis, and Latent Profile Analysis.	Greater out-of-pocket expenditures were typically associated with substandard impacts on the caregivers. Some expenditures in particular, such as the kitchen, bathroom, and scooter were associated with a lower impact. The caregivers with lower impact reported the highest mobility, cognitive, and upper extremity functioning of the patient they care for. Additionally, the highest caregiver impact was influenced by the patient's negative affect and fatigue.
Drivers of Caregiver Impact in Duchenne Muscular Dystrophy: A Cohort Study [9]	566 DMD caregivers	Eligible participants were age 18 or older, able to complete an online questionnaire, and were providing caregiving support to a family member with DMD at least two years old, usually their son.	The association within various psychosocial factors and impact profiles among selected DMD caregivers. The psychosocial factors analyzed incorporated demographic, QOL, life stress, resilience, COVID-related, reserve-building, and cognitive appraisal approach.	Person-reported outcomes (PROs), PROMIS-10 General Health, NeuroQOL Positive Affect and Well-Being, The Ryff Environmental Mastery, Urban Life Stress Inventory, Work Productivity and Activity Impairment measure, USNIH, The CDC Healthy Days Core Module, Current-Reserve-Building Measure, QOL appraisal, and ANOVA.	As the child's disability progresses, the impact experienced by DMD caregivers varies depending on their personal health, environmental mastery, and levels of anxiety. Caregivers who were more significantly affected experienced a worsened impact during the COVID pandemic. Additionally, engagement in passive-media consumption and a cognitive pattern focused on the negative were associated with a worsened impact for caregivers [9]. Stress variables from the high-impact group revealed that difficulty paying bills and hours missed from work was most prominent and worsened compared to the low-impact group.

Future considerations

In terms of future research, studies can account for DMD patients in all age groups. They can indicate the direct and indirect impacts this illness had since the beginning of diagnosis and how it has amplified, and to what extent, over time. Since DMD is a terminal illness, comparing the various impacts of the disease on the caregivers over time will give insight into the effect of the disease progression on the different aspects of their quality of life. Furthermore, focusing on the prevalence concerning the entirety of the male population will reflect the changing age profile of patients with DMD and allow an in-depth analysis of the various burdens caused. 

Based on these studies, we believe it will be beneficial to investigate the various support groups offered to the caregivers of DMD patients, as their psychological well-being plays a vital role in their daily activities. Since this study is international, the supportive programs are unique to each country and cannot be compared, as the number of resources available, government support, and disease frequency play a role in determining the administration of such programs. Thus, further exploring the availability and accessibility of support programs such as therapy will allow caregivers to prioritize their mental health and mitigate the negative impacts taking care of a patient with a terminal illness may induce (e.g., anxiety, depression) [10,12].

## Conclusions

This literature review includes detailed insight regarding the effects of health-related quality of life, psychological well-being, and financial burden on the caregivers of patients with DMD. The studies presented clearly demonstrate the extent to which this terminal illness influences copious aspects of a caregiver's life. With the progression of this disease, evidence shows a decline in HRQoL, which has a directly proportional relationship to one's psychological well-being. More specifically, the psychological well-being of the caregiver also declines due to the anxiety, depression, and financial burden that augments as this disease progresses.
